# Prevalence of cryptosporidiosis among children with diarrhoea under five years admitted to Kosti teaching hospital, Kosti City, Sudan

**DOI:** 10.1186/s12879-021-06047-1

**Published:** 2021-04-14

**Authors:** Abdelhakam G. Tamomh, AbdElhadi M. Agena, Elham Elamin, Mohammed A. Suliman, Mohammed Elmadani, Asmaa B. Omara, Sahar A. Musa

**Affiliations:** 1 Department of Parasitology and Medical Entomology, Faculty of Medical Laboratory Sciences, University of El Imam El Mahdi, 27711 Kosti, Sudan; 2 Department of Haematology, Faculty of Medical Laboratory Sciences, University of El Imam El Mahdi, 27711 Kosti, Sudan; 3 Department of Epidemiology, Faculty of Public and Environmental Health, University of El Imam El Mahdi, 27711 Kosti, Sudan

**Keywords:** Prevalence, Cryptosporidiosis, diarrhoea, Children under 5 years, Kosti

## Abstract

**Background:**

Cryptosporidiosis is a disease caused by infection with an intestinal coccidian parasite Cryptosporidium. Cryptosporidium species are the second leading cause of diarrheal disease and death in children in developing countries. Until now, no data have been available or published on its prevalence among children with diarrhea in Sudan. Therefore, this paper was designed to determine the prevalence rate of Cryptosporidium among children with diarrhea under 5 years who were admitted to Kosti Teaching Hospital.

**Methods:**

A hospital-based cross-sectional study including children under 5 years old admitted to the pediatric section of the hospital between September 2020 and December 2020. A total of one-hundred and fifty stool samples were collected. All stool samples were examined using the modified Ziehl Neelsen (mZN) staining technique and then examined microscopically for Cryptosporidium infection.

**Results:**

A total of 150 children were examined out of which 70 presented with diarrhea. A greater prevalence of 19/70 (27.1%) of Cryptosporidium was observed in children with diarrhea than children without diarrhea 7/80 (8.8%). There was a significant relationship between the prevalence of Cryptosporidium and the presence of diarrhea in children under 5 years in the Kosti Teaching Hospital(*P* < 0.05). It was found that a higher prevalence was registered among children using piped-water sources for drinking.

**Conclusions:**

The overall prevalence of parasite detected was 17.3% among children admitted to Kosti Teaching Hospital. The prevalence rate of the infection among Children with diarrhoea was 27.1%. Studying the prevalence rate of cryptosporidiosis among diarrheic children may predict their health status, leading to a better diagnosis, treatment, and, therefore, patients’ status improvement.

## Background

Cryptosporidium species are single-celled coccidian parasites that cause the diarrheal disease cryptosporidiosis [1–4]. They can be found in soil, food, water, and on surfaces that have been contaminated with fecal matter from infected humans or animals. Cryptosporidiosis may occur via contaminated water used for irrigation or application of agricultural chemicals, floodwater, contacting polluted dump tank or flume water used for post-harvest washing of infected workers [5–9].

Cryptosporidiosis is a worldwide illness caused by the coccidian parasite Cryptosporidium, which infects numerous species of vertebrates, including humans. Watery diarrhea is the most common symptom of the disease. Abdominal cramps, vomiting, nausea, dehydration, and weight loss are other symptoms associated with cryptosporidiosis. The onset of cryptosporidiosis is generally two to ten days after becoming infected with the parasite. Symptoms are usually self-limiting in healthy individuals. Diarrhoea and dehydration may be more severe and possibly life-threatening among individuals with weakened immune systems [10–13].

Cryptosporidiosis is usually diagnosed with microscopic detection of the parasite oocysts, oocyst antigens, or oocyst DNA in stool samples. For the detection of oocysts in stool, the sample must be concentrated using the formalin-ether sedimentation method before microscopic examination. The oocysts of Cryptosporidium in unconcentrated fecal smears can be easily observed by acid-fast or phenol–auramine staining methods [1, 2, 9, 14–18].

About 30–50% of cryptosporidiosis deaths in infants and children occur worldwide. It is the second after rotavirus as a leading cause of diarrhea and deaths among children [1, 9].

In developing countries or low-and-middle-income countries, the parasite was gradually associated with malnutrition and death caused by diarrhea in children [5, 6, 19–23]. Cryptosporidiosis, caused by the *Cryptosporidium parvum*, is of great concern because of its association with economic losses and the public health significance in humans. Over two-hundred water-borne, food-borne, and zoonotic cryptosporidiosis outbreaks have been registered. Cryptosporidium spp. in economically low resource countries are the second cause of diarrheal illness and death in children and remains the only member of the diarrheal diseases for which no consistent and effective therapy is available [7, 10, 11, 24–30].

Sudan is one of these countries, situated in Northeast Africa at the Nile Valley. Most epidemiological studies or surveys have reported and indicated that the infections caused by soil-transmitted helminths, *Schistosoma* spp., food- and water-borne protozoa, and *Plasmodium* spp., are endemic in Sudan [12–14, 17]. However, limited information is available on the prevalence of cryptosporidiosis among children in Sudan, especially children with diarrhea. This study aims to report on the prevalence of Cryptosporidiosis among children with diarrhea attending Kosti Teaching Hospital, White Nile State, Sudan.

## Materials and methods

### Study area

The research was conducted at the pediatric section and clinical laboratory department of Kosti Teaching Hospital, Kosti, Sudan. This Hospital serves customers from Kosti city and its surrounding cities in the State. Kosti city is one of the major cities in Sudan that lies south of Khartoum, the capital of Sudan, and sits at the western bank of the White Nile river opposite Rabak (the capital of the White Nile state) where there is a bridge. The locality is composed of five administrative units. It has been bordered by Eldewiem locality in the north, Rabak locality in the east, Al salam locality in the south, and Tendalty locality in the west. The most essential water resource is rainwater and the White Nile. Most activities are grazing, agriculture, trade, and fishing.

### Study design

This study is a hospital-based cross-sectional study conducted at Kosti Teaching Hospital Laboratory between the first of September and 30th of December, 2020. All participants who were consulted for or hospitalized in the pediatric section were included in the study. A consecutive sampling method was performed and those who met our inclusion criteria and consented to the research were directly included and selected.

### Study population

The participants of this study were children who were 5 years and below and presented at Kosti Teaching Hospital.

### Inclusion criteria

The study was restricted to children who were of the age 5 years and below. They involved those that had diarrhea or without diarrhea and parents/guardians agreed for their children to be part of the study after explaining to them in English and Arabic the objectives of our study.

### Exclusion criteria

Children above 5 years were excluded as well as those children 5 years or below whose parents/guardians did not agree to be part of the study. Additionally, we excluded children whose parents could not give the exact age of their children.

### Ethical considerations

The study was approved by the Institutional Ethics Committee of Faculty of Medical Laboratory Sciences, University of El Imam El Mahdi.

### Data collection

Questionnaires were administered to collect demographic data that included; name, age, sex, clinical symptoms including diarrhea, patient’s residence, presence of toilet facility, and sources of drinking water. This questionnaire was appropriated or adapted from the research of Tombang A N et al. in Cameroon [1].

### Stool sample collection

Parents/guardians of children with gastrointestinal symptoms (with diarrhea or without diarrhea) were given labeled stool containers to collect one stool sample at the time of collection. They were guided on how to collect a suitable amount of stool in the containers and send them to the laboratory as soon as possible.

### Stool sample handling and storage

Suitable gloves were worn to take or handle the containers. The samples were checked for stool quantity and also their physical appearance was recorded. The labels of the containers were also checked and matched to their corresponding questionnaires. Fresh stool samples were kept and preserved at − 20 °C in the freezer of the fridge at the clinical laboratory for investigations at the end of each working day.

### Modified Ziehl Neelsen (mZN) staining technique

The stool samples were taken from the laboratory fridge and put at room temperature before fecal smears were prepared on microscope slides using wooden stick applicators. Then the smears were left on racks to air dry.

### Staining

The slides were put in staining racks for fixation in absolute methanol for 3 min followed by a strong carbol fuchsin stain for 15 min. Then, the slides were rinsed in tap water. A decolorization was made by adding 1% hydrochloric acid alcohol for 15 s and rinsed with tap water. Then, a counterstain of 1% methylene blue was added for 30 s, rinsed well, and left to air dry. The stained slides were investigated and examined microscopically using 40x and 100x objectives and the presence or absence of oocysts was recorded.

### Oocyst determination

Oocysts are round to ovoid, and the size usually ~ 4–6 μm in diameter. They are acid-fast staining. Oocyst is variable in staining. Some oocysts may appear unstained and fully sporulated structures can be found in which a red color staining, crescentic shapes or bodies. The sporozoites can appear within an unstained wall of the oocyst. The quantity of oocysts was determined as 1+ (1-10oocysts/preparation); 2 + (11-50oocysts/preparation); and 3 + (>50oocysts/preparation).

### Statistical analysis

Data was recorded and analyzed using the Chi-square test by a statistical package for social science (SPSS version 21) program. *P* values < 0.05 was considered significant for all statistical analyses.

## Results

### Socio-demographic characteristics of participants

A total of 150 children with ages 5 years old and below were enrolled in this study. The mean age of the children was 46.8 (SD = 17.7) months, and the median age was 60 months. More than half i.e. 85 (56.7%) of the children were males. Also, a total of 137 (91.3%) or 13(8.7%) have latrines or without latrines in their homes respectively (Table 1).

**Table 1:** Demographics data of the studied patients

Characteristics	Frequency (***n*** = 150)	Percentage %
**Age**		
< 5 yrs	63	42
= 5 yrs	87	58
**Gender**		
Male	85	56.7
Female	65	43.3
**Availability of Latrine**		
Yes	137	91.3
No	13	8.7
**Source of drinking water**		
Pipe	116	77.3
Canal	1	0.7
Donkey cart	15	10
Others	18	12

### Diarrhea and stool consistency from children ages 5 years and below in Kosti (*n* = 150)

A total of 70/150 (46.7%) participants were presented with diarrhea (watery/mucoid stool) whereas the remaining 80/150 (53.3%) children were presented with formed/semi-formed stool.

### Prevalence of Cryptosporidium and the presence/absence of diarrhea

Table 2 shows that the age group less than 30 months old recorded a 10/63 (15.8%) and a 16/87 (18.4%) prevalence for the age group equal to 60 months. The participants that presented with diarrhea recorded a 19/70 (27.1%) prevalence while those that did not present with diarrhea recorded a 7/80 (8.8%) prevalence (Table 2).

**Table 2:** Prevalence of Cryptosporidiosis among children according to Age and presence of diarrhoea

Variable	Frequency	Number of Positive	No. of Microscopy positive samples by (%)	No. of Microscopy positive samples within (%)
Age				
< 30 months	63	10	6.7	15.9
= 60 months	87	16	10.7	18.4
Diarrhoea				
yes	70	19	12.7	27.1
No	80	7	4.7	8.8

### Prevalence of Cryptosporidium among children under or equal the ages 5 years in Kosti that presented with diarrhea

Overall, the prevalence of Cryptosporidium among children admitted to Kosti Teaching Hospital was 26/150 (17.3%). A high prevalence of Cryptosporidium (19/70, 27.1%) was observed among children who have diarrhea than in children without diarrhea (7/80, 8.8%). There was a significant relationship (*p* = 0.003) between the prevalence of Cryptosporidium and the presence of diarrhea among children of 5 years and below presented to the Kosti Teaching Hospital (Table 3).

**Table 3:** Relationship of Cryptosporidium among children under 5 years in Kosti with demographics and family data

Variable	No. of stool samples for each categories	No.(100%) positive samples based on categories	Prevalence (17.3%) for each categories	*P- value*
**Age**				
< 5 yrs	63 (42)	10 (38.5)	10 (6.7)	0.688
= 5 yrs	87 (58)	16 (61.5)	16 (10.6)	
**Gender**				
Male	85 (56.7)	18 (69.2)	18 (12)	0.155
Female	65 (43.3)	8 (30.8)	8 (5)	
**Availability of Latrine**				
Yes	137 (91.3)	23 (88.5)	23 (15.3)	0.567
No	13 (8.7)	3 (11.5)	3 (2)	
**Source of drinking water**				
Pipe	116 (77.3)	18 (69.3)	18 (12)	0.367
Canal	1 (0.7)	00	00	
Donkey cart	15 (10)	5 (19.2)	5 (3.3)	
Others	18 (12)	3 (11.5)	3 (2)	
**Diarrhoea**				
Yes	70 (46.7)	19 (73)	19 (12.7)	0.003
No	80 (53.3)	7 (27)	7 (4.6)	

## Discussion

*Cryptosporidium* is among the four major pathogens causing diarrheal diseases in low-and-middle-income countries, especially in children. This parasite is recognized as a highly infectious enteric pathogen and is transmitted mainly by the fecal-oral route [1, 2]. This current study aimed to determine the prevalence rate of Cryptosporidiosis among children with diarrhea admitted to Kosti Teaching hospital, Kosti city, White Nile state in Sudan. One-hundred and fifty diarrhoeic stool samples were examined using first, direct wet preparation and formal ether concentration technique and second, examined using modified ZN staining technique.

In our present findings, the overall prevalence rate of cryptosporidiosis was 26 (17.3%) among children with diarrhea admitted to the pediatrics emergency section at Kosti Teaching Hospital using the modified ziehl neelsen technique. This result shows a higher prevalence compared to the results reported in Tanzania, Cameroon, Rwanda, and Benin [1–3, 6] with the prevalence of 10.4, 8.93, 9.4, and 5.8% respectively. A higher prevalence rate has also been found in low-and-middle-income countries with high average rainfall such as Nigeria [5], with 38.3%. Our result is in line with the result obtained in residents in rural areas of the White Nile state in Sudan (13.3%) and near to that result obtained in Iraq (21.92%) [4, 7]. This prevalence rate is probably justified by fact that Kosti is a city with improved access to drinkable water, latrine use, less fecal contamination rates, thus limiting the occurrence of parasitosis in general and cryptosporidiosis in particular. Also, this low prevalence may be explained by the methodology, i.e. using microscopy can be less sensitive than PCR.

Our relevant analysis explained that the prevalence of cryptosporidiosis among diarrhoeic children was a higher increase in males 18 (12%) than in females 8 (5%). But there was no significant difference (*p* > 0.05). The reason for these differences is not clear since they have the same exposure at the crawling stage but probably be due to the fact that females show less activity level and are mostly home. This result is comparable to the results obtained in Iraq [4].

In the current findings, a high detection or recovery of cryptosporidium parasite was found among the age group of five years 16 (10.7%) than the age under five years old 10 (6.7%). This finding is in agreement with research carried out in Germany, and Cameroon [1, 8] and by the Centers for Disease Control (CDC) in 2020 [9] where a greater prevalence rate was observed among children of the ages of 5 years than those under 5 years. In this age, the high prevalence is maybe related to the fact that this age group is usually in danger of diarrhea, for the reason that those basal hygiene activities are unknown or not respected and paired with the fact that the immune system of their body is not sophisticated [9]. This high prevalence rate of children with ages equal to 5 years could be because schooling ages in Sudan are usually around five years old. Also, in this age group, this prevalence can be inferred from the fact that the crest of parasitism occurs at the ages of kindergarten and primary schools when community games involve touching contaminated and dirty soil. Perhaps, it is for this reason, that this current research was conducted in Kosti, an urban and mostly civilian area with a multiracial population, where children find better care than children in rural areas where the rates of malnutrition are greater.

Further investigations expressed the prevalence was high among children who consumed pipeline water 18 (12%) than the other sources of water. Despite pipeline water with a purification system which is the main source of water for most populations in Kosti city, this high prevalence rate may be related sometimes to the stopping of water purification system and it may be associated with economic improvement. The risk of using contaminated bare hands for feeding children may enable the transmission of foodborne diseases like Cryptosporidiosis from infected adults to children. These practices can generate big chances for the ingestion of contaminated food and water with oocysts shed from Cryptosporidium-infected individuals. This elaborates why Cryptosporidium is ranked 5th among the most important foodborne parasites globally [2, 5, 7, 30].

## Conclusion

The overall prevalence of Cryptosporidiosis among children who are 5 years and below who were admitted at Kosti Teaching Hospital was 17.3%. The Prevalence rate among children that have diarrhea was 27.1%. Children whose parents were using pipe water for drinking registered a higher prevalence. Additionally, there is an association between Cryptosporidium and diarrhea among children who are 5 years and below admitted with diarrhea in Kosti. Our findings clearly explained that Cryptosporidium is an essential causative agent of diarrhea for children in Kosti. It is necessary to build or improve more water purification systems and sanitation facilities to help the population of this area to access clean drinking water for preventing cryptosporidium infection. Health education and personal hygiene programs should be held to teach people how to avoid the infection.

### Limitation of the study

Freezing and thawing of stool samples may lead to fragmentation of oocysts and undercounting. Staining of Spores and artifacts with mZN stain may lead to false-positive results for untrained eyes. Additionally, a too thick smear prepared may not adequately destain and therefore, false-negative results may occur.

## Data Availability

The data used to support the findings of this study are available from the corresponding author upon request.
